# Effects of nurse-led symptom management in chronic myeloid malignancies: a randomized trial

**DOI:** 10.1007/s00520-025-09230-1

**Published:** 2025-02-15

**Authors:** Maja Pedersen, Maria Torp Larsen, Brian Thomas Kornblit, Emma Ove Dahl, Kirsten Lomborg, Anders Tolver, Mary Jarden

**Affiliations:** 1 Department of Hematology, Centre for Cancer and Organ Diseases, Copenhagen University Hospital, Rigshospitalet, Blegdamsvej 9, 2100 Copenhagen, Denmark; 2 Department of Clinical Medicine, University of Copenhagen, Blegdamsvej 3B, 2200 Copenhagen, Denmark; 3 Department of Mathematical Sciences, Data Science Laboratory, University of Copenhagen, Universitetsparken 5, 2200 Copenhagen, Denmark; 4 Department of Clinical Research, Steno Diabetes Center Copenhagen, Borgmester Ib Juuls Vej 83, 2730 Herlev, Denmark

**Keywords:** Hematological malignancies, Nurse-led, Symptom management, Patient-reported outcomes, HM-PRO, Quality of life

## Abstract

**Purpose:**

Chronic hematological malignancies progress slowly, potentially manifesting symptoms spanning months to years. HM-PRO is developed as a comprehensive clinical tool for assessing symptoms in hematology. The aim was to investigate the effect of a nurse-led systematic approach to symptom identification and management using HM-PRO in outpatient care in patients with chronic hematological malignancies.

**Methods:**

This is a randomized trial including 94 patients to investigate an intervention comprising (1) HM-PRO data collection, (2) HM-PRO assessment guided by an algorithm, and (3) nurse-led tailored symptom management. The control arm received standard follow-up care. The primary outcome was change in QoL. Secondary outcomes were change in prevalence of physical and psychological symptoms.

**Results:**

A statistically significant difference in QoL change scores over time favored the intervention (diff. 10.3; *p* = .04). For secondary endpoints, a significant between group difference in change over time for severity scores was observed in fatigue (diff. − 13.6; *p* = .003), overall symptom burden (diff. − 0.7 points; *p* = .029), emotional functioning (diff. 10.0; *p* < .0001), and anxiety (diff. − 2.5; *p* = .001).

**Conclusion:**

A 12-month nurse-led symptom management intervention within hematology significantly improved QoL, emotional functioning, fatigue, anxiety, and overall symptom burden over time. This is the first randomized trial investigating nurse-led clinical application of the HM-PRO questionnaire providing knowledge on the efficacy of systematic symptom management in clinical practice. This study highlights both the pivotal role of nurses and multidisciplinary support and the inherent value of tailored symptom management.

**Trial registration:**

Clinical trial registration number: NCT04757545 (02/12/2021).

## Introduction

Advances in hematological cancer treatment have improved clinical outcomes and survival rates in recent decades [1]. Improved survival has, in turn, led to a growing number of patients receiving treatment and long-term monitoring in outpatient settings.

Hematological malignancies manifest differently. Acute leukemia and lymphoma have an acute onset, while multiple myeloma (MM), chronic myeloid leukemia (CML), myelodysplastic syndrome (MDS), and myeloproliferative neoplasms (MPN) have a chronic course. Consequently, treatment modalities range from aggressive chemotherapeutic regimens to lower dose maintenance regimes or a “watch and wait” approach [2, 3]. Chronic hematological malignancies gradually progress, potentially accumulating symptoms over extended periods, spanning months to years [3, 4]. Therefore, prolonged monitoring in an outpatient setting is an important strategy to help chronic malignant patients endure the impact of symptoms, ability to work, and independence in daily life [5–7]. Patients report various symptoms with fatigue being the most prevalent [8, 9]. Furthermore, difficulty sleeping, drowsiness, lack of focus, pain and stress are also frequently reported in patients with chronic hematological malignancies [8]. Consequently, many patients have a diminished quality of life (QoL) [9, 10].

Utilization of patient reported outcomes (PRO) has the potential to alleviate symptoms and identify unmet patient needs [11]. In solid tumors, systematic integration of PRO in clinical settings has reduced symptoms without requiring prolonged clinical contact [11, 12]. However, collecting PROs alone may not improve patient health outcomes, as healthcare professionals need to act on this knowledge [13]. Therefore, complementing PRO assessments with evidence-based symptom management is imperative, requiring a multidisciplinary approach [13]. This approach to managing symptoms in cancer is beneficial, with nurses specializing in cancer care often playing a pivotal role in assisting patients with symptom management [14, 15].

Symptoms associated with hematological malignancies can vary in type, duration, and severity depending on the diagnose and treatment. Generic cancer research questionnaires are primarily designed for research rather than clinical purposes [16, 17]. Hematological Malignancy Patient-Reported Outcome (HM-PRO) is a questionnaire developed for clinical use tailored specifically to hematological malignancies [18]. HM-PRO is designed as a comprehensive symptom assessment and support tool for routine follow-up care, comprising aspects of physical, social, emotional behavior and well-being, and well-being affected by eating and drinking, and 18 individual signs and symptoms related to disease or treatment [18]. To the best of our knowledge, the HM-PRO questionnaire has not been previously used as a part of a clinical intervention in a randomized trial.

We hypothesized that nurse-led symptom identification and management, facilitated by a disease specific and clinically developed PRO (HM-PRO) could reduce symptom burden while preserving QoL. The aim of this study was to investigate the effect of a systematic nurse-led approach using HM-PRO in outpatient care in patients with chronic hematological malignancies.

## Methods

The methods adhere to the recommendations in the CONSORT statement for randomized trials [19, 20]. The study protocol was registered with the Regional Ethics Committee for the Capital Region of Denmark (20070444) and received approval by the Danish Protection Agency (P-2020–1085).

### Design

This was a two-arm, single-center randomized controlled trial (RCT).

### Participants, recruitment, and randomization procedures

Participants eligible for this study were adults ≥ 18 years old diagnosed with clonal cytopenia of unknown significance (CCUS), CML, MDS, or MPN. Participants were eligible for inclusion six months after diagnosis or later if in stable condition, as assessed by their primary hematologist. A stable condition was defined as one in which patients could be monitored through telephone consultations, supplemented with four annual blood samples. Exclusion criteria included non-proficiency in Danish and/or cognitive/psychiatric challenges preventing participation in a clinical trial.

Recruitment of participants took place in the outpatient clinic at the Department of Hematology at a large University Hospital from February 2021 to January 2022. Participants were identified, screened, and informed of the study by their primary hematologist. Upon consent, the primary investigator approached, informed, and recruited participants for the study. All participants were provided with information about the study, and after a period of consideration, the participants provided written consent to participate.

Following inclusion and baseline assessment, participants were stratified by sex and randomly allocated to either the intervention or control group, with random allocation sequence generated by a computer.

While participants and nurses were not blinded to group allocation, the intervention nurses had no knowledge of or contact with the control group participants. The statistician responsible for the statistical analysis was blinded to group allocation.

### Intervention group

The intervention group received a nurse-led systematic symptom management intervention in conjunction with standard care.

### Intervention: nurse-led systematic symptom management

The symptom management model serves as the theoretical foundation for the intervention, providing a framework for (1) symptom experience, (2) management strategies, and (3) outcome evaluation, which are influenced by three domains: a personal domain, a health and illness domain, and an environmental domain [21]. This model provides a multifactorial approach by not only identifying symptoms but also understanding the complex and dynamic interplay of symptoms, demographic and contextual factors, and strategies to intervene [21]. The elements of the symptom management model driving the hypothesized change are enhanced symptom experience, symptom status, and symptom management to reduce symptoms and maintain QoL in patients with chronic hematological malignancies.

**Fig. 1:**
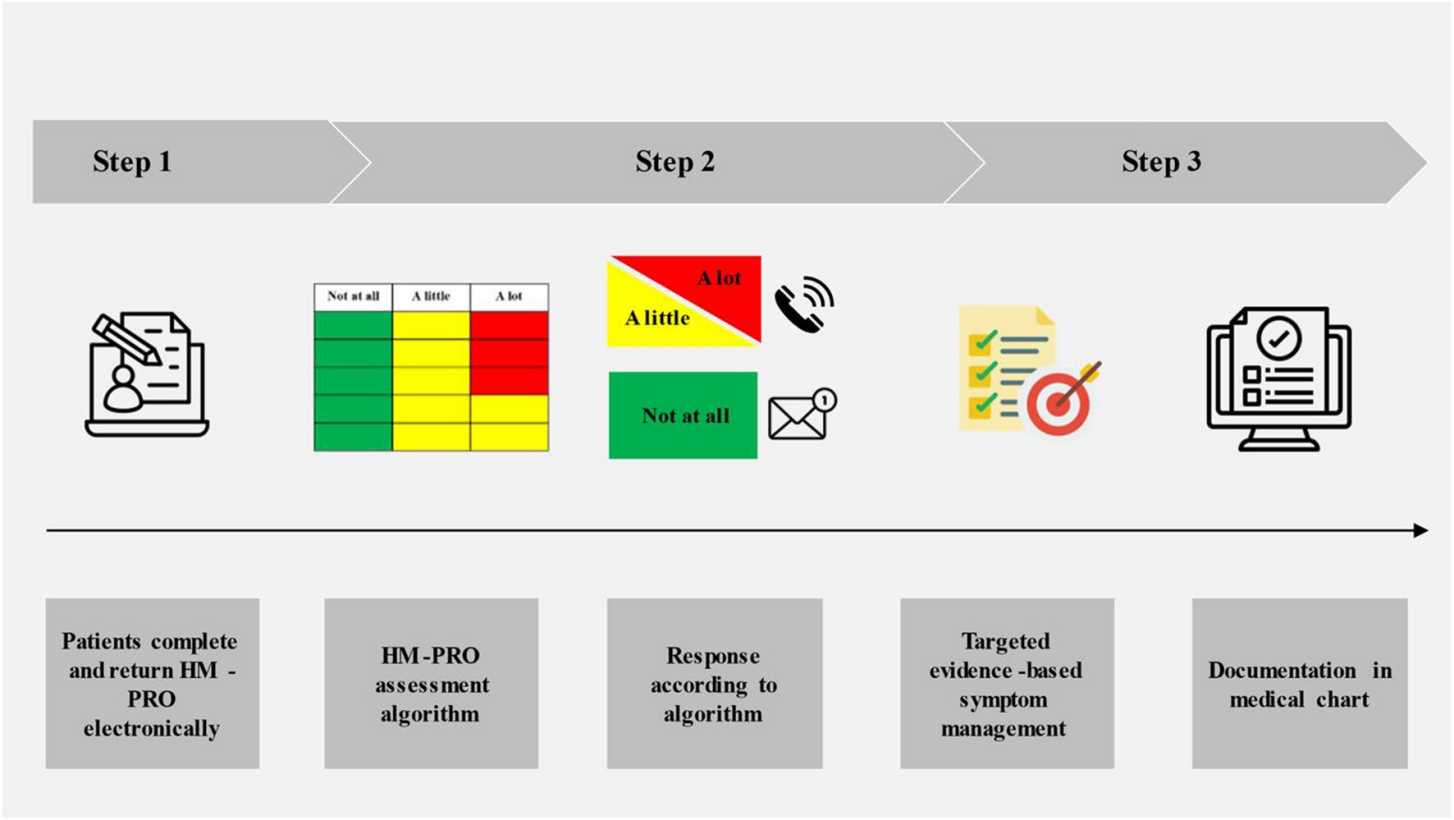
The symptom management intervention. Abbreviations: HM-PRO, Hematological Malignancy Patient-Reported Outcome

Based on the theoretical underpinning, a symptom management intervention was developed by a project group comprising physicians, nurses, nurse specialists, researchers, and patient representatives. The intervention consisted of three steps: (1) HM-PRO response: 1 week before a scheduled nurse-led conversation at 1, 6, and 12 months, participants received the HM-PRO questionnaire to complete and return electronically, with a reminder sent after 5 days if needed; (2) HM-PRO assessment algorithm: nurses analyzed the HM-PRO responses using an algorithm that was developed in the project group to assess and rate symptom severity, guiding systematic and actionable management of symptoms; and (3) nurse-led tailored symptom management conversations guided by the algorithm and a symptom management manual. Participants either received a telephone conversation including nurse-led symptom management or they were sent a personalized text message through the electronic health care system (EPIC) in the absence of moderate or severe symptoms. A symptom management manual ensured structured and evidence-based management of symptoms [21, 22]. The symptom management intervention is illustrated in Fig. 1.

The intervention was conducted between February 2021 and February 2023 by three experienced hematology nurses (9–16 years of experience). Two of the nurses participated in the development phase of the intervention, while one nurse was involved during the study. Workshops including the PI, hematologists, and nurses were held to refine the algorithm, test and adjust the symptom management manual, and organize administrative tasks.

### Control group

The control group received standard care consisting of a scheduled annual phone consultation with a hematologist to evaluate medical status and symptom control as well as four blood samples per year.

### Data collection and time points

Questionnaires were electronically distributed to participants via REDCap, a platform to secure data for research purposes [23]. The data in REDCap consisted of (1) demographic information provided by the participants (sex, age, civil status, working status), (2) HM-PRO intervention data, (3) research endpoint data, and (4) clinical data retrieved from medical journals (diagnosis, time of diagnosis, clinical contact during the intervention period and content of consultations).

The primary endpoint was the between-group difference in QoL measured by the European Organization for Research and Treatment of Cancer Quality of Life Questionnaire Core 30 (EORTC QLQ-C30), specifically the global health domain, from baseline to 12 months [24]. EORTC-QLQ-C30 consists of five functional scales: a physical, cognitive, emotional, role and social scale, three symptom scales (fatigue, pain, and nausea and vomiting), a global health domain, single items assessing symptoms, and perceived financial impact of cancer [24]. The scoring scale range from 1 to 100, with high functional scale/QoL scores indicating a high level of functioning/QoL. In contrast, on the symptom scale, a higher scores represent a high level of symptomatology [25].

Secondary endpoints were changes in symptoms of depression and anxiety measured by Hospital Anxiety and Depression Scale (HADS) [26] and changes in symptom core and interference measured by The M.D. Anderson Symptom Inventory (MDASI) [27]. HADS is a 14-item scale divided into two subscales, with a higher score indicating poorer mental outcomes [26]. MDASI includes 19 items measuring 13 core symptoms and six items related to interference in daily living. The scale ranges from 1 to 10, where a high number indicates a worsening in symptoms [27].

Endpoint data were collected at baseline, 6 months and post testing at 12 months after concluding the symptom management intervention.

Selected data from intervention, including data from the HM-PRO questionnaires, participation in the intervention, and content of the consultations, will be presented in the result section.

### Statistical analysis

The sample size calculation was based on a two-sample *t*-test, assuming a minimal clinically relevant between-group difference of 10 and an estimated within group standard deviation (SD) of 15 on changes from baseline to 12 months for QoL. To achieve a statistical power of 0.80, maintain a type 1 error rate of 5%, and account for an expected dropout rate of 20%, we determined that a sample size of 45 patients per group would be adequate for this study [28–30]. Demographic and clinical variables were reported as means and ranges for quantitative variables and as numbers and percentages for categorial variables. Raw means and standard deviations were reported for primary and secondary outcomes. A linear mixed effects model with random effect of the patient and fixed effects of treatment, time, and their interaction was fitted to all outcomes. Estimates for within-group changes from baseline to post testing, as well as between group differences of changes, were extracted from the model together with *t*-tests. A *p*-value less than 0.05 was considered statistically significant. However, due to the large number of secondary outcomes and the high risk of reporting false positive results, the results should be viewed as exploratory. The statistical analysis was conducted using R.

## Results

**Fig. 2:**
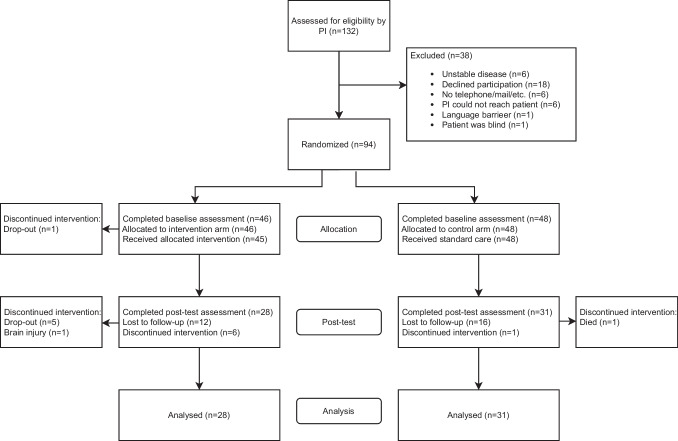
Flowchart

Initially, 343 patients were screened by a hematologist from ambulatory patient lists, followed by the primary hematologist assessing patients for inclusion. Subsequently, the PI invited 132 eligible patients, and out of these, 94 (72%) patients provided consent for participation and were enrolled. After baseline assessment, participants were randomly allocated to the intervention group (*n* = 46) or the control group (*n* = 48). The flow of participants in the study is illustrated in Fig. 2.

Baseline characteristics, as illustrated in Table 1, showed a median age of 64.7 years in the intervention group and 63.9 years in the control group. In the intervention group, 50.0% were female, while 56.2% in the control group were female. The predominant diagnosis was MPN (76%), followed by CML (18%), MDS (3%), and CCUS (3%).

**Table 1:** Demographic and medical characteristics of the participants

**Variables**	Intervention group (*n* = 46) *n* (%)	Control group (*n* = 48) *n* (%)
Sex Female Male	23 (50.0) 23 (50.0)	27 (56.2) 21 (43.8)
Age, mean (range)	64.7 (30–79)	63.9 (37–86)
Diagnose MPN CML MDS CCUS	36 (78.3) 8 (17.4) 0 (0) 2 (4.3)	35 (72.9) 9 (18.8) 3 (6.2) 1 (2.1)
Time since diagnose, years, mean (range)	7.7 (0.8–32)	5.2 (0.6–18)
Civil status Widow (er) Married Cohabiting Single No information from patient	4 (8.7) 28 (60.9) 2 (4.3) 11 (23.9) 1 (2.2)	6 (12.5) 25 (52.1) 4 (8.3) 13 (27.1) 0 (0)
Employment status Employed full time Employed part time < 32 h/week Sick leave Retired/pension Early retired Not working, self-selected No information from patient	16 (34.8) 2 (4.3) 1 (2.2) 23 (50) 1 (2.2) 1 (2.2) 2 (4.3)	15 (31.2) 3 (6.2) 1 (2.1) 26 (54.2) 2 (4.2) 1 (2.1) 0 (0)
Education Primary school Short education Medium education Higher education No information from patient	5 (10.9) 12 (26.1) 17 (37) 11 (23.9) 1 (2.2)	7 (14.6) 12 (25) 12 (25) 17 (35.9) 0 (0)

*CCUS*, clonal cytopenia of unknown significance; *CML*, chronic myeloid leukemia; *MDS*, myelodysplastic syndrome; *MPN*, myeloproliferative neoplasia

### Primary endpoint

The primary endpoint was the between group-difference in QoL at 12 months. A statistically significant difference in change scores over time favored the intervention group (between group diff. 10.3 (SE 5.0) *p* = 0.040). Results are shown in Table 2.

**Table 2:** Quality of life and symptoms at baseline, 6 and 12 months, within and across groups

	Intervention group, raw mean (SD)			In-group change, mean (SE)^a, b^	*p*-value^b^	Control group, raw mean (SD)			In-group change, mean (SE)^a, b^	*p*-value^b^	Between-group differences in change, mean (SE)^c^	*p*-value^b,c^
	Baseline (*n* = 46)	6 months (*n* = 28)	12 months (*n* = 28)			Baseline (*n* = 48)	6 months (*n* = 35)	12 months (*n* = 31)				
EORTC-QLQ-C30												
Global health status	72.4 (20.9)	73.5 (24.9)	77.4 (22.3)	6.2 (3.6)	.200	72.7 (20.0)	72.6 (21.5)	68.6 (22.3)	− 4.1 (3.4)	.460	10.3 (5.0)	.040
Physical functioning	87.4 (13.6)	84.3 (15.7)	86.0 (18.1)	0.6 (2.1)	.952	86.4 (17.0)	84.4 (18.0)	83.7 (17.5)	− 3.6 (1.9)	.162	4.2 (2.8)	.142
Emotional functioning	86.9 (17.3)	89.9 (16.9)	90.8 (15.1)	4.6 (2.8)	.216	82.8 (19.5)	82.7 (17.8)	75.2 (24.6)	− 5.3 (2.6)	.108	10.0 (3.8)	< .0001
Cognitive functioning	89.3 (14.7)	89.9 (13.1)	89.9 (16.6)	2.0 (2.5)	.703	85.8 (16.1)	87.1 (17.7)	84.4 (15.5)	− 0.8 (2.4)	.942	2.8 (3.5)	.421
Social functioning	91.5 (14.9)	89.3 (16.5)	89.3 (19.9)	2.1 (3.1)	.766	90.6 (15.3)	91.0 (18.2)	88.7 (16.3)	2.6 (2.9)	.646	0.5 (4.2)	.912
Role functioning	85.9 (19.6)	80.4 (24.9)	82.7 (26.6)	2.1 (3.5)	.821	82.6 (24.8)	82.4 (23.6)	79.0 (26.9)	4.1 (3.4)	.454	1.9 (4.9)	.694
Fatigue	27.9 (24.8)	29.0 (26.4)	24.6 (24.8)	− 6.0 (3.2)	.149	24.1 (19.9)	27.0 (22.9)	32.3 (25.4)	7.6 (3.0)	.038	− 13.6 (4.4)	.003
Nausea and vomiting	3.7 (11.7)	3.6 (8.3)	4.8 (10.0)	0.7 (1.6)	.908	2.1 (6.6)	2.4 (5.9)	3.8 (11.2)	1.2 (1.5)	.691	− 0.6 (2.2)	.793
Pain	17.8 (20.5)	17.9 (26.0)	17.3 (21.5)	− 1.9 (4.2)	.896	23.3 (28.9)	22.4 (26.5)	23.7 (30.4)	0.9 (4.0)	.971	− 2.8 (5.8)	.631
Dyspnea	13.3 (22.9)	14.3 (24.7)	17.9 (26.4)	1.5 (3.4)	.897	20.1 (26.4)	21.0 (30.3)	25.8 (28.2)	3.0 (3.2)	.625	− 1.5 (4.7)	.753
Insomnia	23.0 (25.5)	17.9 (24.8)	19.1 (21.1)	− 3.8 (4.6)	.691	20.1 (24.5)	21.0 (23.0)	29.0 (35.6)	8.2 (4.4)	.148	− 12.0 (6.3)	.061
Appetite loss	5.2 (15.8)	6.0 (13.0)	2.4 (8.7)	− 1.2 (3.0)	.922	5.6 (14.3)	10.5 (19.4)	11.8 (25.2)	6.9 (2.9)	.044	− 8.2 (4.2)	.054
Constipation	8.2 (16.1)	6.0 (14.0)	10.7 (18.3)	3.1 (3.0)	.562	8.3 (16.1)	9.5 (22.3)	11.8 (23.7)	1.4 (2.9)	.877	1.7 (4.2)	.685
Diarrhea	11.1 (21.3)	10.7 (20.4)	15.5 (21.2)	2.6 (4.1)	.846	9.7 (20.6)	15.7 (28.7)	9.68 (23.1)	− 0.5 (3.9)	.990	2.8 (5.6)	.623
Financial difficulties	6.7 (18.3)	3.6 (13.9)	4.8 (11.9)	− 1.1 (2.4)	.898	1.4 (6.7)	3.8 (10.8)	4.30 (14.3)	4.1 (2.3)	.180	− 5.1 (3.3)	.124
HADS												
Anxiety	4.3 (3.9)	3.7 (3.9)	3.4 (3.5)	− 1.1 (0.5)	.079	3.9 (2.8)	3.7 (3.1)	5.4 (3.8)	1.4 (0.5)	.015	− 2.5 (0.7)	.001
Depression	2.6 (2.9)	2.7 (2.5)	3.2 (3.3)	0.0 (0.5)	.998	3.3 (3.3)	3.4 (3.4)	4.2 (3.4)	1.0 (0.5)	.143	− 1.0 (0.7)	.171
MDASI												
Symptoms; core	1.1 (1.3)	1.10 (1.1)	1.0 (1.0)	− 0.3 (0.2)	.396	1.5 (1.4)	1.8 (1.5)	1.2 (1.7)	− 0.4 (0.2)	.171	− 0.7 (0.3)	.029
Symptoms, interference	1.1 (1.4)	1.6 (1.5)	1.17 (1.7)	− 0.1 (0.4)	.952	1.7 (2.2)	2.2 (2.5)	2.5 (2.6)	0.6 (0.4)	.196	− 0.7 (0.5)	.149

^a^In-group changes from baseline to 12 months

^b^Linear mixed model adjusted for baseline

^c^Between-group differences in change from baseline to 12 months

Abbreviations: *EORTC-QLQ-C30*, European Organization for Research and Treatment of Cancer Quality of Life Questionnaire Core 30; *HADS*, Hospital Anxiety and Depression Scale; *MDASI*, M.D. Anderson Symptom Inventory

### Secondary endpoint

Fatigue prevalence measured by EORTC-QLQ-C30 remained unchanged in the intervention group (diff. − 6.0 (SE 3.2); *p* = 0.149), but significantly increased in the control group (diff. 7.5 (SE 3.0); *p* = 0.038). Consequently, a significant difference in fatigue prevalence over time favoring the intervention group (diff. − 13.6 (SE 4.4); *p* = 0.003) was observed. The between-group difference in change scores for overall symptom burden related to core symptoms was statistically significant, with a difference of − 0.7 points (SE 0.3); *p* = 0.029. In contrast, the difference between changes of symptom interference over time was non-significant.

Emotional functioning measured by EORTC-QLQ-C30 significantly changed between-groups, favoring the intervention group (diff. 10.0 (SE 3.8); *p* < 0.0001). However, within-group differences were non-significant. For HADS, anxiety symptoms significantly worsened over time in the control group (diff. 1.4 (SE 0.5); *p* = 0.015), resulting in a statistically significant difference in the development over time when comparing the two groups (diff. − 2.5 (SE 0.7); *p* = 0.001). In the intervention group, the level of symptoms of anxiety remained stable (diff. − 1.1 (SE 0.5); *p* = 0.079). In depression, no significant differences were found.

Total clinical contacts regardless of type (face to face, telephone, or written, but excluding symptom management telephone conversations) were balanced between the intervention group (*n* = 106) and the control group (*n* = 105).

### Intervention, participation, and content

The response rates for the HM-PRO questionnaires at 1, 6, and 12 months were 93.5%, 80.0%, and 80.0%, respectively. Adherence to the subsequent nurse-led symptom management at 1, 6, and 12 months was 80.4%, 62.2%, and 72.5%, respectively as shown in Table 3.

**Table 3:** Completion of HM-PRO and nurse-led symptom management according to the algorithm

Time points (*n* = eligible patients at time points)	HM-PRO completion *n* (%)	Received symptom management *n* (%)	Type of symptom management contact according to algorithm		
			Telephone *n* (%)	Written *n* (%)	Missed *n* (%)
1 month (*n* = 46)	43 (93.5)	37 (80.4)	34 (79)	3 (7)	6 (14)
6 months (*n* = 45)	36 (80.0)	28 (62.2)	25 (70)	3 (8)	8 (22)
12 months (*n* = 40)	32 (80.0)	29 (72.5)	27 (85)	2 (6)	3 (9)

*Abbreviations*: *HM-PRO*, Hematological Malignancy Patient-reported outcome

The HM-PRO questionnaire data showed emotional and physical well-being as the most affected domains. The intervention nurses consistently adhered to the symptom management protocol, as indicated by clinical record data. The topics addressed in the conversations aligned with participant responses obtained from the HM-PRO questionnaire. While most conversations were conducted by nurses, there were also instances necessitating a multidisciplinary approach. Other collaborators, primarily the primary hematologist (*n* = 9), were engaged. Additionally, nurses offered several participants referral for rehabilitation follow-up in the municipality. Out of 23 participants that were offered municipal follow-up, 14 accepted. Some participants declined (*n* = 6), while others were already committed to rehabilitation activities (*n* = 3).

## Discussion

This randomized trial investigated the effect of a systematic approach to nurse-led symptom management using HM-PRO questionnaire in outpatient care in patients with chronic hematological malignancies. The results favored the intervention, indicating that a 12-month symptom management intervention was effective. The difference in QoL demonstrated in this study is considered clinically relevant, equivalent to a medium effect [30], and the levels of QoL are consistent with findings in other studies including chronic hematological malignancies [31, 32]. Prior randomized trials have observed enhanced QoL in patients with hematological malignancies who underwent a PRO-based intervention [33, 34]. While the specific components of these interventions may vary, a common objective was to positively impact QoL and enhance symptom management, aligning with the aim of our study.

Changes in fatigue scores measured by EORTC-QLQ-C30 indicated a medium difference in mean scores, implying that the difference in fatigue may have clinical significance for the patients [30]. This finding is supported by previous randomized trials investigating the effect of non-pharmacological interventions within hematology, showing improvements in fatigue-related outcomes [34, 35].

Over time, patients in the control group exhibited a worsening of symptoms of anxiety, as measured by HADS, and compared to the intervention group, the changes over time from baseline to 12 months were both statistically significant and clinically relevant [36].

HM-PRO data from the intervention group consistently highlighted the impact on participants’ emotional well-being, making it a focus of discussion in the subsequent symptom management conversations. At 12-month post-testing, the longitudinal changes in emotional functioning measured by EORTC-QLQ-C30 significantly favored the intervention group. This suggests a connection between the participants’ responses on the HM-PRO questionnaire, the topics discussed during the symptom management consultations, and the results derived from the post-test endpoint data.

This intervention’s “dosage” is low and non-invasive in daily life, tailored to each patient’s needs rather than following a one-size-fits-all delivery model. However, HM-PRO responses (*n* = 8) reporting no or few symptoms, who received only written correspondence guided by the algorithm were unexpectedly low (Table 3), emphasizing the need for nurse-led symptom management for chronic hematological malignancies. In future trials, there is potential to further refine the algorithm to ensure precise delivery of the intervention to other patient groups within hematology.

Hematology nurses were responsible for the symptom management; however, certain conversations required multidisciplinary involvement. The multidisciplinary nature of addressing symptoms in cancer is acknowledged in previous research [37]. Specialized cancer care nurses are recognized for their pivotal role in guiding and coordinating patients’ pathways within the healthcare system, and a symptom science colloquium from NINR, ONS, and NCI emphasizes the distinctive role of oncology nurses and nursing researchers in exploring and investigating symptom science and QoL [14, 15, 38]. The results of this study support the effectiveness of a nurse-led intervention, showing positive effects on both QoL and symptom outcomes. Conducted in the clinic by designated hematology nurses, this study provides informative and applicable results, underscoring the potential value of nurses taking on this task with implications for clinical practice.

The tailored approach enabled prioritization of symptom management in relevant patients, promoting individualized health interventions. A limitation of this model of care is the inter-individual variability of symptoms, as symptoms tend to fluctuate over time [39]. The limited frequency of collecting PRO data in this intervention, only three times in 12 months, can be subject to criticism. Comparing weekly PRO measurements to daily measurements diminishes the opportunity for timely intervention because symptoms tend to fluctuate [40]. However, this study includes patients with chronic hematological malignancies, distinguishing it from more acute and aggressive cancer trajectories. Non-pharmacological interventions for hematological patients have been mainly investigated in those with acute diseases such as leukemia, with sparse evidence on chronic hematological conditions despite their recognized symptom burden [3, 4, 41]. Hence, this study has the potential to inform health care professionals about a group of patients less prioritized in previous research.

This study has several strengths. A randomized trial, the gold standard in medical research, was utilized to investigate the effect of an intervention, and validated questionnaires were used to collect endpoint data. Furthermore, the intervention was conducted in a real-world setting within the clinic. The HM-PRO questionnaire, developed for clinical purposes, was a cornerstone in the intervention, and to the best of our knowledge, this is the first randomized trial to investigate clinical application of HM-PRO. Finally, in standard care, nurses do not typically interact with this patient group, eliminating contamination between intervention and control group treatment.

The study has some limitations. First, excluding patients without technical skills could decrease diversity and affect external validity. Interventions using PRO data frequently require that patients have technical skills [42]. Second, both the intervention and research assessments rely on collecting PRO data from patients. These PRO questionnaires might appear similar, and patients with fatigue might find the quantity of questions overwhelming, potentially lowering response rates. Using several questionnaires increases the risk of duplication of questions, which could result in an unnecessary burden for the patients [43]. Third, the number of participants lost to follow-up could impact the results, as reasons for not adhering to the intervention or post assessment at 6 and 12 months remain unknown. The number of lost to follow-up was approximately balanced between intervention and control group. Adherence to the intervention declined to 62.2% at 6 months and 72.2% at 12 months. The decrease at 6 months was primarily attributed to organizational challenges stemming from the department’s involvement in a significant merger, a nationwide nurse strike, and a prolonged emphasis on COVID-19. Fourth, the study population mainly consisted of patients diagnosed with MPN, reducing the generalizability of the findings. Fifth, the medical treatment might have affected the burden of symptoms causing a potential bias between groups. Sixth, the statistical power of this study was designed to detect changes in the primary outcome, QoL, over time. Consequently, caution is advised when interpreting the secondary outcomes due to the potential risk of type II error. However, these findings might serve as a foundation for generating hypotheses in future research.

## Conclusion

A 12-month nurse-led symptom management intervention in patients with chronic hematological malignancy has proven effective, demonstrating significant benefits in QoL. Symptom relief remained consistent, in the intervention group, whereas the control group experienced an escalation in certain symptoms. The intervention group showed significant improvement from baseline to 12 months, particularly in emotional functioning, fatigue, anxiety, and overall symptom burden compared to the control group.

This is the first randomized trial to investigate nurse-led clinical application of the HM-PRO questionnaire. The findings provide valuable insights for healthcare professionals and future researchers regarding the efficacy of incorporating HM-PRO into a clinical pragmatic randomized trial. The results are not only informative but also applicable for clinical practice, emphasizing the value of tailored symptom management, where nurses play a crucial role in helping patients in effectively managing their symptoms.

## Data Availability

No datasets were generated or analysed during the current study.

## References

[1] Pulte D, Jansen L, Brenner H (2020) Changes in long term survival after diagnosis with common hematologic malignancies in the early 21st century. Blood Cancer J 10:56. 10.1038/s41408-020-0323-4 32404891 10.1038/s41408-020-0323-4 PMC7221083

[2] Rodriguez-Abreu D, Bordoni A, Zucca E (2007) Epidemiology of hematological malignancies. Ann Oncol 18(Suppl 1):i3–i8. 10.1093/annonc/mdl443 17311819 10.1093/annonc/mdl443

[3] Kaifie A, Isfort S, Gattermann N et al (2016) Health care setting and severity, symptom burden, and complications in patients with Philadelphia-negative myeloproliferative neoplasms (MPN): a comparison between university hospitals, community hospitals, and office-based physicians. Ann Hematol 95:1399–1410. 10.1007/s00277-016-2730-y 27334946 10.1007/s00277-016-2730-y

[4] Geyer HL, Kosiorek H, Dueck AC et al (2017) Associations between gender, disease features and symptom burden in patients with myeloproliferative neoplasms: an analysis by the MPN QOL International Working Group. Haematologica 102:85–93. 10.3324/haematol.2016.149559 27540137 10.3324/haematol.2016.149559 PMC5210236

[5] Manitta V, Zordan R, Cole-Sinclair M et al (2011) The symptom burden of patients with hematological malignancy: a cross-sectional observational study. J Pain Symptom Manage 42:432–442. 10.1016/j.jpainsymman.2010.12.008 21477979 10.1016/j.jpainsymman.2010.12.008

[6] Boyes AW, Clinton-McHarg T, Waller AE et al (2015) Prevalence and correlates of the unmet supportive care needs of individuals diagnosed with a haematological malignancy. Acta Oncol 54:507–514. 10.3109/0284186X.2014.958527 25238282 10.3109/0284186X.2014.958527

[7] Mehnert A (2011) Employment and work-related issues in cancer survivors. Crit Rev Oncol Hematol 77:109–130. 10.1016/j.critrevonc.2010.01.004 20117019 10.1016/j.critrevonc.2010.01.004

[8] Geyer H, Mesa RA (2017) Approach to MPN symptom assessment. Curr Hematol Malig Rep 12:381–388. 10.1007/s11899-017-0399-5 28942516 10.1007/s11899-017-0399-5 PMC8148891

[9] Allart-Vorelli P, Porro B, Baguet F et al (2015) Haematological cancer and quality of life: a systematic literature review. Blood Cancer J 5:e305. 10.1038/bcj.2015.29 25909835 10.1038/bcj.2015.29 PMC4450328

[10] Tolstrup Larsen R, Tang LH, Brochmann N et al (2018) Associations between fatigue, physical activity, and QoL in patients with myeloproliferative neoplasms. Eur J Haematol 100:550–559. 10.1111/ejh.13048 29464777 10.1111/ejh.13048

[11] Berry DL, Hong F, Halpenny B et al (2014) Electronic self-report assessment for cancer and self-care support: results of a multicenter randomized trial. J Clin Oncol 32:199–205. 10.1200/JCO.2013.48.6662 24344222 10.1200/JCO.2013.48.6662 PMC3887477

[12] Howell D, Molloy S, Wilkinson K et al (2015) Patient-reported outcomes in routine cancer clinical practice: a scoping review of use, impact on health outcomes, and implementation factors. Ann Oncol 26:1846–1858. 10.1093/annonc/mdv181 25888610 10.1093/annonc/mdv181

[13] Mooney K, Berry DL, Whisenant M, Sjoberg D (2017) Improving cancer care through the patient experience: how to use patient-reported outcomes in clinical practice. Am Soc Clin Oncol Educ Book 37:695–704. 10.1200/EDBK_175418 28561689 10.1200/EDBK_175418

[14] Charalambous A, Wells M, Campbell P et al (2018) A scoping review of trials of interventions led or delivered by cancer nurses. Int J Nurs Stud 86:36–43. 10.1016/j.ijnurstu.2018.05.014 29960894 10.1016/j.ijnurstu.2018.05.014

[15] de Leeuw J, Larsson M (2013) Nurse-led follow-up care for cancer patients: what is known and what is needed. Support Care Cancer 21:2643–2649. 10.1007/s00520-013-1892-6 23828397 10.1007/s00520-013-1892-6

[16] Goswami P, Khatib Y, Salek S (2019) Haematological malignancy: Are we measuring what is important to patients? A systematic review of quality-of-life instruments. Eur J Haematol 102:279–311. 10.1111/ejh.13203 30556217 10.1111/ejh.13203

[17] Goswami P, Oliva EN, Ionova T et al (2020) Quality-of-life issues and symptoms reported by patients living with haematological malignancy: a qualitative study. Ther Adv Hematol 11:204062072095500. 10.1177/2040620720955002 10.1177/2040620720955002 PMC7549153 33101618

[18] Goswami P, Oliva EN, Ionova T et al (2020) Development of a novel hematological malignancy specific patient-reported outcome measure (HM-PRO): content validity. Front Pharmacol 11:209. 10.3389/fphar.2020.00209 32210809 10.3389/fphar.2020.00209 PMC7066982

[19] Schulz KF, Altman DG, Moher D, CONSORT Group (2010) CONSORT 2010 statement: updated guidelines for reporting parallel group randomised trials. BMJ 340:c332. 10.1136/bmj.c332 20332509 10.1136/bmj.c332 PMC2844940

[20] Boutron I, Moher D, Altman DG et al (2008) Extending the CONSORT statement to randomized trials of nonpharmacologic treatment: explanation and elaboration. Ann Intern Med 148:295–309. 10.7326/0003-4819-148-4-200802190-00008 18283207 10.7326/0003-4819-148-4-200802190-00008

[21] Dodd M, Janson S, Facione N et al (2001) Advancing the science of symptom management. J Adv Nurs 33:668–676. 10.1046/j.1365-2648.2001.01697.x 11298204 10.1046/j.1365-2648.2001.01697.x

[22] Fortin AH (2019) Smith’s patient centered interviewing: an evidence-based method, 4th edn. McGraw-Hill Education, New York

[23] Harris PA, Taylor R, Thielke R et al (2009) Research electronic data capture (REDCap)–a metadata-driven methodology and workflow process for providing translational research informatics support. J Biomed Inform 42:377–381. 10.1016/j.jbi.2008.08.010 18929686 10.1016/j.jbi.2008.08.010 PMC2700030

[24] Aaronson NK, Ahmedzai S, Bergman B et al (1993) The European Organization for Research and Treatment of Cancer QLQ-C30: a quality-of-life instrument for use in international clinical trials in oncology. J Natl Cancer Inst 85:365–376. 10.1093/jnci/85.5.365 8433390 10.1093/jnci/85.5.365

[25] Fayers P, Bottomley A, EORTC Quality of Life Group, Quality of Life Unit (2002) Quality of life research within the EORTC-the EORTC QLQ-C30. European Organisation for Research and Treatment of Cancer. Eur J Cancer 38(Suppl 4):S125-133. 10.1016/s0959-8049(01)00448-8 11858978 10.1016/s0959-8049(01)00448-8

[26] Zigmond AS, Snaith RP (1983) The hospital anxiety and depression scale. Acta Psychiatr Scand 67:361–370. 10.1111/j.1600-0447.1983.tb09716.x 6880820 10.1111/j.1600-0447.1983.tb09716.x

[27] Cleeland CS, Mendoza TR, Wang XS et al (2000) Assessing symptom distress in cancer patients: the M.D. Anderson Symptom Inventory Cancer 89:1634–1646. 10.1002/1097-0142(20001001)89:7%3c1634::aid-cncr29%3e3.0.co;2-v 10.1002/1097-0142(20001001)89:7<1634::aid-cncr29>3.0.co;2-v 11013380

[28] Musoro JZ, Coens C, Sprangers MAG et al (2023) Minimally important differences for interpreting EORTC QLQ-C30 change scores over time: a synthesis across 21 clinical trials involving nine different cancer types. Eur J Cancer 188:171–182. 10.1016/j.ejca.2023.04.027 37257278 10.1016/j.ejca.2023.04.027

[29] Pahl A, Wehrle A, Kneis S et al (2020) Whole body vibration training during allogeneic hematopoietic cell transplantation-the effects on patients’ physical capacity. Ann Hematol 99:635–648. 10.1007/s00277-020-03921-x 31970448 10.1007/s00277-020-03921-x PMC7060160

[30] Cocks K, King MT, Velikova G et al (2011) Evidence-based guidelines for determination of sample size and interpretation of the European Organisation for the Research and Treatment of Cancer Quality of life questionnaire core 30. J Clin Oncol 29:89–96. 10.1200/JCO.2010.28.0107 21098316 10.1200/JCO.2010.28.0107

[31] Johnsen AT, Tholstrup D, Petersen MA et al (2009) Health related quality of life in a nationally representative sample of haematological patients. Eur J Haematol 83:139–148. 10.1111/j.1600-0609.2009.01250.x 19284418 10.1111/j.1600-0609.2009.01250.x PMC2730555

[32] Efficace F, Iurlo A, Patriarca A et al (2021) Validation and reference values of the EORTC QLQ-CML24 questionnaire to assess health-related quality of life in patients with chronic myeloid leukemia. Leuk Lymphoma 62:669–678. 10.1080/10428194.2020.1838509 33153355 10.1080/10428194.2020.1838509

[33] Sagari A, Ikio Y, Imamura N et al (2018) Effect of occupation-based interventions in patients with haematopoietic malignancies undergoing chemotherapy: a pilot randomised controlled trial. Hong Kong J Occup Ther 31:97–105. 10.1177/1569186118818680 30643497 10.1177/1569186118818680 PMC6322108

[34] Jim HSL, Hyland KA, Nelson AM et al (2020) Internet-assisted cognitive behavioral intervention for targeted therapy-related fatigue in chronic myeloid leukemia: results from a pilot randomized trial. Cancer 126:174–180. 10.1002/cncr.32521 31553815 10.1002/cncr.32521 PMC6906223

[35] Bryant AL, Coffman E, Phillips B et al (2020) Pilot randomized trial of an electronic symptom monitoring and reporting intervention for hospitalized adults undergoing hematopoietic stem cell transplantation. Support Care Cancer 28:1223–1231. 10.1007/s00520-019-04932-9 31222392 10.1007/s00520-019-04932-9 PMC6923608

[36] Lemay KR, Tulloch HE, Pipe AL, Reed JL (2019) Establishing the minimal clinically important difference for the hospital anxiety and depression scale in patients with cardiovascular disease. J Cardiopulm Rehabil Prev 39:E6–E11. 10.1097/HCR.0000000000000379 30489438 10.1097/HCR.0000000000000379

[37] El-Jawahri A, LeBlanc TW, Kavanaugh A et al (2021) Effectiveness of integrated palliative and oncology care for patients with acute myeloid leukemia: a randomized clinical trial. JAMA Oncol 7:238–245. 10.1001/jamaoncol.2020.6343 33331857 10.1001/jamaoncol.2020.6343 PMC7747042

[38] Von Ah D, Cooley ME, Bailey DE Jr et al (2022) Oncology nursing symptom science: overview of the NINR, ONS, and NCI symptom science colloquium. Oncol Nurs Forum 49:105–112. 10.1188/22.ONF.105-112 35191901 10.1188/22.ONF.105-112

[39] King-Kallimanis BL, Bhatnagar V, Horodniceanu EG et al (2022) Timing is everything: the importance of patient-reported outcome assessment frequency when characterizing symptomatic adverse events. Clin Trials 19:267–273. 10.1177/17407745221093935 35575012 10.1177/17407745221093935

[40] Daly B, Nicholas K, Flynn J et al (2022) Analysis of a remote monitoring program for symptoms among adults with cancer receiving antineoplastic therapy. JAMA Netw Open 5:e221078. 10.1001/jamanetworkopen.2022.1078 35244701 10.1001/jamanetworkopen.2022.1078 PMC8897754

[41] Rossau HK, Kjerholt M, Brochmann N et al (2022) Daily living and rehabilitation needs in patients and caregivers affected by myeloproliferative neoplasms (MPN): a qualitative study. J Clin Nurs 31:909–921. 10.1111/jocn.15944 34231273 10.1111/jocn.15944

[42] van Egdom LSE, Oemrawsingh A, Verweij LM et al (2019) Implementing patient-reported outcome measures in clinical breast cancer care: a systematic review. Value in Health 22:1197–1226. 10.1016/j.jval.2019.04.1927 31563263 10.1016/j.jval.2019.04.1927

[43] Kluetz PG, Chingos DT, Basch EM, Mitchell SA (2016) Patient-reported outcomes in cancer clinical trials: measuring symptomatic adverse events with the National Cancer Institute’s Patient-Reported Outcomes Version of the common Terminology Criteria for Adverse Events (PRO-CTCAE). Am Soc Clin Oncol Educ Book 35:67–73. 10.1200/EDBK_159514 27249687 10.1200/EDBK_159514

